# Co-exposure to arsenic and nickel induces oxidative stress and mineral imbalance, impairing male reproductive parameters in Wistar rats

**DOI:** 10.1007/s10534-026-00797-8

**Published:** 2026-03-05

**Authors:** Thainá Iasbik-Lima, Luiz Otávio Guimarães-Ervilha, Tayná Bolsam da Silva, Renê Chagas da Silva, Mariana Machado-Neves

**Affiliations:** 1 Departamento de Biologia Geral, Universidade Federal de Viçosa, Av. P.H. Rolfs, S/N DBG, ECS333, Campus Universitário, Viçosa, Minas Gerais CEP 36570-900 Brazil; 2 Departamento de Medicina Veterinária, Universidade Federal de Viçosa, Viçosa, Minas Gerais CEP 36.570-900 Brazil; 3 Departamento de Física, Universidade Federal de Viçosa, Viçosa, Minas Gerais CEP 36.570-900 Brazil

**Keywords:** Reproductive toxicity, Testis, Epididymis, Sperm

## Abstract

Simultaneous exposure to multiple toxic metals is a common scenario in environmental contamination. Among these metals, arsenic and nickel are widely distributed pollutants with well-established toxic effects. However, their combined impact on male reproductive physiology and micromineral homeostasis remains poorly understood. This study evaluated the effects of subchronic co-exposure to arsenic (1 mg L^−1^) and nickel (7 mg L^−1^) in drinking water for 70 days on the hypothalamus, testis, and epididymis of adult Wistar rats. Exposed rats showed arsenic and nickel retention in all analyzed tissues, low serum testosterone levels, and alterations in the proportion of chemical elements, including copper, zinc, calcium, iron, and manganese. These findings were associated with an antioxidant enzyme dysregulation and high generation of protein carbonyls and nitric oxide in testicular and epididymal tissues, respectively. Consequently, the testes of co-exposed rats exhibited alterations in stereological and morphometric parameters, low daily sperm production, and initial histological changes in response to the toxic metals’ presence. The hypothalamus exhibited focal areas of neuronal degeneration, especially in co-exposed rats. The epididymis of co-exposed animals presented focal areas of inflammatory infiltrates and germ cells within the luminal duct, with an acceleration of sperm transit time. Spermatozoa from all exposed rats showed low motility and high morphological abnormalities in the head, while the co-exposure increased the occurrence of midpiece and tail defects. These findings highlight the synergistic toxicity of arsenic and nickel on the male reproductive system after subchronic exposure, with a direct role of bioaccumulation and trace element dysregulation.

## Introduction

Exposure to heavy metals is a global concern due to their potential for bioaccumulation and the deleterious effects they can cause on human health, including hormonal dysfunctions, oxidative stress, and damage to the nervous, cardiovascular, and reproductive systems (Han et al. 2020; Mitra et al. 2022; Jomova et al. 2025). Among these metals, arsenic and nickel stand out, as they are widely distributed in the environment and frequently associated with environmental contamination (Genchi et al. 2020; Fatoki and Badmus 2022; Su et al. 2023; Rizwan et al. 2024). Although arsenic and nickel occur naturally in the Earth’s crust, anthropogenic activities, such as industrial processes, mining, and environmental disasters, primarily contribute to their harmful accumulation in ecosystems, contaminating soil, water, and food (Ibrahim et al. 2006; Orloff et al. 2009; Kumar et al. 2021).

Exposure to arsenic is associated with a series of adverse health effects, including liver toxicity, neurotoxicity, and endocrine disruption (Jomova et al. 2011). In the male reproductive system, arsenic compromises testicular function by reducing testosterone production and altering the expression of enzymes involved in steroidogenesis (Seif et al. 2021). Histologically, the testis of animals exposed to this metalloid exhibits atrophy of the seminiferous tubules, vacuolization of the germinal epithelium, and increased apoptosis of germ cells (Biswas et al. 2023; Guimarães-Ervilha et al. 2025). The epididymis, in turn, suffers disorganization of the epithelium and a reduction in the concentration of mature sperm (Machado-Neves 2022). In addition, arsenic induces oxidative stress in the testis and epididymis that, in turn, leads to lipid peroxidation and DNA damage in sperm cells, compromising their viability and motility (Souza et al. 2019; Biswas et al. 2020).

Nickel also exerts toxic effects on male reproductive health, being a metal capable of acting as an endocrine disruptor (Kong et al. 2021; Maric et al. 2023). Nickel exposure results in sperm impairment, such as low viability, high DNA fragmentation, and high morphological defects (Sen et al. 2019). At the histological level, the testes of animals exposed to this transitional metal presented degeneration of the seminiferous tubules, disorganization of Sertoli cells, detachment of immature germ cells, and disruption of testosterone production influenced by a reduction in the Leydig cell population (Jargar et al. 2012; Iftikhar et al. 2023). In the epididymis, nickel accumulation elicited tissue inflammation, altering the luminal microenvironment necessary for sperm maturation and storage (Yang et al. 2021). Moreover, nickel promotes an imbalance in the oxidative profile, reducing the activity of antioxidant enzymes and increasing the levels of reactive oxygen species, which can contribute to structural and functional damage to the male reproductive organs (Kong et al. 2019; Adedara et al. 2019a).

The male reproductive system is particularly vulnerable to toxic agents, as it depends on hormonal and antioxidant balance to maintain its functionality (Aitken and Roman 2008; O’Flaherty 2019). A hormonal cascade begins in the hypothalamus, with the hypothalamic-pituitary–gonadal axis responsible for regulating the release and feedback of follicle-stimulating hormone (FSH) and luteinizing hormone (LH) by the pituitary gland (Koysombat et al. 2023). These hormones support the functions of the testis, which play a central role in testosterone production and spermatogenesis (Li et al. 2024). Spermatogenesis occurs within the seminiferous tubules, where germ cells undergo mitotic and meiotic divisions until they differentiate into sperm (Hess and Franca 2008). After being released from the testicles, the immature sperm pass through the epididymis, acquiring progressive motility and fertilization capacity through interactions with proteins in the epididymal fluid (Robaire and Hilton 2015). This sperm maturation process is essential for male fertility, and any change in the physiology of the testicle or epididymis can compromise sperm quality and reduce reproductive capacity (Sullivan and Mieusset 2016).

Although the toxicity of heavy metals has been widely studied, most research focuses on a single exposure rather than exposure to a metal mixture, neglecting the environmental reality in which contaminants rarely occur alone. Combined exposure to metals such as arsenic and nickel can generate antagonistic or synergistic effects, exacerbating damage to exposed organisms (Andrade et al. 2015). Events such as the environmental disaster in Minas Gerais, Brazil, resulted in the contamination of soil and water by a mixture of metals, including arsenic and nickel, reinforcing the need for research on the combined effects of these substances on body organs (Parente et al. 2021). Thus, this study aimed to validate the toxic impact of isolated arsenic and nickel and to investigate the effects of exposure to the mixture of these two chemical elements on the hypothalamus, testis, epididymis, and sperm of adult Wistar rats. To this end, we assessed the homeostasis of microminerals, metal retention, oxidative profile, and histological changes in male organs, serum testosterone levels, and sperm parameters resulting from this potentially toxic interaction.

## Materials and methods

### Animals and ethics statement

Seventy-day-old male Wistar rats (*n* = 40; ~ 310 g) were provided by the Central Animal Facility of the Federal University of Viçosa (UFV). The animals were housed individually in polypropylene cages with wood shaving bedding under controlled temperature (21 °C) and photoperiod (12 h light/dark cycles). All rats had access to rat chow (Nuvilab®) and drinking water ad libitum. This study strictly followed the recommendations of the National Council for the Control of Animal Experimentation (CONCEA). All experimental procedures were reviewed and approved by the UFV Animal Use Ethics Committee (CEUA process number 45/2021).

### Experimental design

After a ten-day acclimatization period, sexually mature animals were randomly assigned to four experimental groups (*n* = 10/group). Control rats (Control group) received 0.9% saline. Males from arsenic and nickel groups were exposed to 1 mg L^−1^ of the metalloid (As group) and 7 mg L^−1^ of the transition metal (Ni group), respectively. These doses were calculated considering the metal element content in the salt’s sodium arsenite (NaAsO₂; Sigma Aldrich Co., St. Louis, MO) and nickel chloride hexahydrate (NiCl_2_·6H_2_O; Sigma Aldrich Co., St. Louis, MO). Rats from the fourth group were co-exposed to a mixture of arsenic at 1 mg L^−1^ and nickel at 7 mg L^−1^ (As + Ni group). These salts were chosen because they are highly soluble in water and are found contaminating groundwater available for humans and animals (Souza et al. 2016; El-Naggar et al. 2021). The solutions were prepared daily to minimize metal oxidation and were provided ad libitum in the drinking water for 70 days. Water consumption was monitored daily to calculate the milligrams of arsenic and nickel per body weight (BW) per day (mg metal/kg BW/ day) following Souza et al. (2018). This exposure period encompasses a subchronic exposure that spans the duration of spermatogenesis (~ 52 days) in rats and the sperm transit time in their epididymis (~ 10 days), thereby establishing a sperm reserve within the cauda epididymis (Machado-Neves 2022).

The doses of arsenic and nickel provided correspond to 100 times the maximum concentrations tolerated in drinking water (arsenic at 0.01 mg L^−1^ and nickel at 0.07 mg L^−1^, respectively) by the World Health Organization (WHO 2011). Assuming that 7 mg L^−1^ of nickel and 1 mg L^−1^ of arsenic in rats, when normalized for body surface area (Reagan-Shaw et al. 2008), correspond to, respectively, 1.13 mg kg^−1^ and 0.162 mg kg^−1^. These two concentrations may be encountered in groundwater from countries worldwide, representing ecotoxicological and environmental relevance in exposure scenarios involving high concentrations of arsenic and nickel (Heikkinen et al. 2002; Smedley and Kinniburgh 2002; Mukherjee et al. 2006; Rahman et al. 2009; Chow et al. 2021).

### Euthanasia, organ collection, and biometry

After 70 days of the experiment, the rats (*n* = 10/group) were weighed and fasted for 10 h. Afterward, the animals were euthanized by deep anesthesia (xylazine 10 mg kg^−1^ BW intraperitoneal [i.p.] and ketamine 150 mg kg^−1^ BW i.p.) followed by cardiac puncture. Blood was collected for hormonal analysis. The cranial vault was opened, and the brain was removed and subsequently dissected to obtain the hypothalamus. The testes and epididymides were removed, dissected, and weighed to determine absolute and relative weights; the latter was calculated based on the final body weight. The hypothalamus was split longitudinally; one half was fixed to perform histological analysis, and the other half was frozen at − 80 °C for antioxidant enzyme activity and trace element content. The left testis and epididymis were fixed for histological analysis, and the right ones were frozen for quantifying antioxidant enzyme activity, oxidative metabolites, mineral content, daily sperm production, and sperm transit time in the epididymis. The epididymis was also processed to assess sperm parameters.

### Determination of serum testosterone levels

Blood samples (*n* = 6/group) were centrifuged at 2000×*g* for 15 min. The serum obtained was used to quantify serum testosterone levels using chemiluminescence at the Laboratório Diagnósticos do Brasil (https://www.diagnosticosdobrasil.com.br/) (Carvalho et al. 2022). The results were expressed in ng dL^−1^.

### Micromineral analysis in the hypothalamus, testis, and epididymis

The proportion of trace elements in the hypothalamus, the testis, and the cauda region of the epididymis (*n* = 6/group) was estimated in frozen organ fragments (Guimarães-Ervilha et al. 2023). Briefly, the fragments were dried in an oven at 60 °C for 96 h, coated with carbon (Quorum Q150 T, East Grinstead, West Sussex, United Kingdom), and analyzed under a scanning electron microscope (JEOL, JSM-6010LA) equipped with energy-dispersive X-ray spectroscopy (EDS). A proportion of arsenic (As), nickel (Ni), magnesium (Mg), potassium (K), calcium (Ca), manganese (Mn), iron (Fe), copper (Cu), zinc (Zn), and selenium (Se) was obtained. Results were expressed as mean percentage values of each element relative to the total detected elemental content in the analyzed tissue.

### Antioxidant enzyme activity in the hypothalamus, testis, and epididymis

Frozen fragments of the hypothalamus, testis, and cauda epididymis (100 mg; *n* = 6/group) were homogenized in 1 mL of phosphate-buffered saline (PBS, pH 7.4) and centrifuged at 15,000×*g* for 10 min at 4 °C. The supernatant was used to determine the activity of antioxidant enzymes and oxidative stress markers, and the pellet was used to determine protein carbonyl levels. Superoxide dismutase (SOD) activity was assessed by the pyrogallol method based on the enzyme’s ability to catalyze the superoxide (O_2_^−^) reaction (Marklund and Marklund 1974). Catalase (CAT) activity was measured as described by Aebi (1984) with modifications. The supernatant was incubated with the substrate (50 mM PBS, pH 7.4, with 20 mM H_2_O_2_). Subsequently, ammonium molybdate was added to stop the reaction, and the values ​​were calculated from a standard curve using a known concentration of H_2_O_2_. Glutathione S-transferase (GST) activity was evaluated according to Habig et al. (1974) by monitoring the formation of the 1-chloro-2, 4-dinitrobenzene (CDNB)–glutathione conjugate. Absorbance was measured at 340 nm at 30 and 90 s intervals. The molar extinction coefficient used for CDNB is ε340 = 9.6 mmol × L^−1^ × cm^−1^. Bradford’s method (1976) normalized the results to total protein levels, and enzyme activity was expressed as units per milligram of protein. Total antioxidant potential was determined using the iron-reducing antioxidant power (FRAP) method following Benzie & Strain (1996). The FRAP assay consisted of a colorimetric measurement of the reduction of the ferric-tripyridyl triazine (Fe^3+^-TPTZ) complex to ferrous tripyridyl triazine (Fe^2+^-TPTZ) by sample antioxidants. The results were expressed as μM Fe^2+^.

### Markers of oxidative and nitrosative stress in the testis and epididymis

Byproducts of oxidative and nitrosative stress were measured in the testis and cauda epididymis, except for the hypothalamic tissue, due to the scarce material. Nitric oxide (NO) levels were measured indirectly in the supernatant by quantifying nitrite/nitrate levels according to the Griess methodology (Tsikas 2007). The results were expressed in μmol L^−1^. Lipid peroxidation status was determined by examining tissue malondialdehyde (MDA) levels, where an aliquot of the supernatant was incubated in a water bath with thiobarbituric acid reactive substances (TBARS) (Buege & Aust 1978). MDA levels were expressed as nmol per milligram of protein. Protein peroxidation was analyzed by quantifying protein carbonyls (PC) in the pellet using the 2,4-dinitrophenylhydrazine (DNPH) method (Levine et al. 1990). The results were expressed in nmol mL^−1^, based on the molar extinction coefficient of ε370 = 22 mmol × L^−1^ × cm^−1^. The analyses were performed using an ELISA microplate reader (Multiskan GO, Thermo Scientific).

### Histological processing and histopathology in the hypothalamus, testis, and epididymis

The hypothalamus, left testis, and left epididymis (*n* = 6/group) were immersed in Karnovsky’s fixative solution (2.5% glutaraldehyde, 4% paraformaldehyde in 0.1 M sodium phosphate buffer, pH 7.2) for 24 h. After the fixation period, the epididymis was fragmented, separating the initial segment, caput, corpus, and cauda regions. The fragments of the hypothalamus, testis, and epididymal regions were dehydrated in an increasing series of ethanol (70, 80, 90%, and absolute) and embedded in 2-hydroxyethyl methacrylate (Historesin®, Leica Microsystems, Nussloch, Germany). Semi-serial sections of 3 μm thickness were obtained using a rotary microtome (RM 2255, Leica, Nussloch, Germany), using a distance of 30 μm between sections. The hypothalamus was stained with hematoxylin/eosin, while the testis and epididymis were stained with toluidine blue/sodium borate (1%). The slides were mounted with Entellan® (Merck, Germany).

The organs were qualitatively evaluated under light microscopy (Olympus CX40, Tokyo, Japan). The evaluation was based on predefined morphological criteria described by Souza et al. (2019) and Kempinas and Klinefelter (2015). In the hypothalamus, normal histology was characterized by preserving neuronal architecture with well-organized cells and no signs of degeneration. The presence of degenerating cells was considered a pathological alteration. In the testis, normal histology includes well-organized seminiferous tubules, with continuous germinal epithelium and germ cells arranged in regular layers. Seminiferous tubules presenting vacuolization of the epithelium, tubular degeneration, and germ cells in the lumen were considered abnormal (Souza et al. 2019). The histology of epididymal regions was analyzed according to ductal and interductal organization, including lumen with no abnormal/germ cells, intact epithelium, and interstitium without inflammatory signs. The presence of germ cells in the lumen, epithelial vacuolization, and interstitium inflammatory infiltrates were examples of pathological changes (Kempinas and Klinefelter 2015).

### Testicular histomorphometry

Digital images of testicular parenchyma (*n* = 6/group) were acquired using a photomicroscope (Olympus BX-53, Tokyo, Japan) and analyzed using Image-Pro Plus 4.5® software (Media Cybernetics, Silver Spring, MD, USA). The average tubular diameter, height of the epithelium, and luminal diameter were obtained in 30 transverse sections of seminiferous tubules per animal, using the tubules with the most circular form (Guimarães-Ervilha et al. 2021). Measurements were expressed in μm. The nuclear diameter (μm) of 30 Leydig cells was recorded per animal.

### Testicular stereology

Stereological analyses of the testis (*n* = 6/group) were performed using Image-Pro Plus 4.5® software (Media Cybernetics, Silver Spring, MD, USA). To estimate the proportion of tubular and intertubular compartments, we used a grid with 266 intersections in 10 images obtained using a microscope (Olympus BX-53, Tokyo, Japan) equipped with a digital camera (Olympus DP73, Tokyo, Japan), totaling 2660 points per animal. Correspondence points were recorded in the tubular compartment (tunica propria, seminiferous epithelium, and lumen) and intertubule. The percentage of points in each component was calculated using the formula: volumetric proportion (%) = (number of points in the structure of interest/2660 total points) × 100. Each testicular component’s volume (mL) was calculated using the formula: % of the structure of interest × testicular parenchyma volume/100. The volumetric proportion of intertubular components was obtained by counting 1000 points on images of the intertubular compartment per animal. Points were counted in the Leydig cell nucleus and cytoplasm, lymphatic space, blood vessels, macrophages, and connective tissue. The percentage of points in each component was calculated by the formula: volumetric proportion of the intertubular compartment (%) = (number of points in the intertubular component/1000 total points) × 100. The absolute volume of each intertubular component (mL) was calculated by multiplying its volumetric proportion by the parenchymal volume of the testis and dividing by 100 (Russell et al. 1990).

The nuclear diameter of Leydig cells allows us to calculate the volume occupied by their nucleus and cytoplasm, as well as the total volume of the cells. Leydig nucleus volume was obtained using the mean nuclear diameter and the formula 4/3 *πR*^3^, where *R* = nuclear diameter/2. Leydig cytoplasm volume was estimated using the formula: percentage of cytoplasm × nuclear volume obtained/nuclear percentage. Cell volume was estimated by adding nuclear and cytoplasmic volumes (Russell et al. 1990; Dias et al. 2020). These values were expressed in μm^3^. Based on the calculations presented by Dias et al. (2020), it was possible to obtain the total number of Leydig cells in the testis by dividing the total volume occupied by Leydig cells in the testicular parenchyma by the volume of an individual Leydig cell.

### Daily sperm production, sperm number, and sperm transit time in the epididymis

The tunica albuginea of the right testis (*n* = 6/group) was removed, and the testicular parenchyma was weighed and homogenized for 3 min in 5 mL of saline-triton solution (0.9% NaCl and 0.05% Triton X-100). The homogenate was diluted tenfold and used to count the number of spermatids resistant to homogenization in each sample using Neubauer chambers (four fields per animal). Daily sperm production was assessed by dividing the number of spermatids per testis by 6.1, the number of days that stage 19 spermatids resistant to homogenization are present in the seminiferous epithelium (Robb et al. 1978). Similarly, the caput/corpus and cauda portions of the right epididymis were homogenized (1:20), and sperm were counted as described for the testis. Sperm transit time through the epididymis was calculated by dividing the number of sperm in each portion of the epididymis by the daily sperm production (Robb et al. 1978).

### Sperm analysis

Freshly dissected portions of the cauda epididymis (*n* = 6/group) were cut into small pieces, and 500 μL of a Tris-citric-fructose solution (3.025 g Tris, 1.7 g citric acid, 1.25 g fructose in 100 mL distilled water) was added to allow sperm release. Aliquots of this fluid were collected for sperm analysis. Ten μL were placed between a slide and a coverslip previously heated to 37 °C, and sperm motility was examined under a phase contrast microscope (L-1000B, Bioval, São Paulo, Brazil) at 400 × magnification. One hundred cells were evaluated per animal and classified as motile or nonmotile (Souza et al. 2019). Still, sperm morphology was assessed using 50 µL of epididymal fluid fixed in 100 µL of 4% buffered formaldehyde. Samples were placed on glass slides and analyzed under a phase contrast microscope at 1000 × magnification. Two hundred sperm were evaluated for normal and abnormal morphology (Filler 1993). Results were expressed as percentages.

### Statistical analysis

The normality of the results was assessed by the Shapiro–Wilk test. Percentage data were first converted using the arcsine square root transformation before assessing normality. Parametric data were submitted to the one-way analysis of variance test (*one-way* ANOVA), followed by the post hoc Tukey’s test*.* Also, nonparametric data (percentage of connective tissue and Leydig cells) were submitted to the Kruskal–Wallis test, followed by Dunn’s post hoc test. Differences between groups were significant when p < 0.05. Statistical analysis and graphics were performed using GraphPad Prism 7.0 (GraphPad Software Inc., San Diego, CA, USA). Results were expressed as mean ± standard deviation of the mean (SD).

## Results

### Metal consumption, biometric parameters, and serum testosterone levels

Rats in the As and Ni groups consumed 0.12 ± 0.01 mg As/Kg BW and 0.78 ± 0.05 mg Ni/Kg BW per day. The animals co-exposed to the metals simultaneously ingested 0.11 ± 0.01 mg As/Kg BW and 0.79 ± 0.06 mg Ni/Kg BW per day. Biometric results, including body, testis, epididymis, albuginea, and parenchyma weights, did not differ between control and metal-exposed groups (p > 0.05; Table 1). Isolated and combined exposure to arsenic and nickel caused a reduction in serum testosterone levels when compared to control rats (p < 0.05; Table 1). This reduction was more remarkable in rats from the As + Ni group (p < 0.05; Table 1).

**Table 1:** Biometry of body and male organs and serum testosterone levels from adult Wistar rats exposed to arsenic (As) and nickel (Ni) in drinking water for 70 days

Parameters	Control	1 mg L^−1^ As	7 mg L^−1^ Ni	1 mg L^−1^ As + 7 mg L^−1^ Ni
Initial body weight (g)	321.10 ± 33.53^a^	331.40 ± 9.82^a^	341.0 ± 19.72^a^	328.90 ± 25.96^a^
Final body weight (g)	399.3 ± 25.11^a^	436.3 ± 19.80^a^	438.2 ± 33.88^a^	436.80 ± 44.35^a^
Testis (g)	1.86 ± 0.15^a^	1.91 ± 0.15^a^	1.83 ± 0.14^a^	1.86 ± 0.08^a^
Testis (g/100 g)	0.48 ± 0.02^a^	0.46 ± 0.05^a^	0.44 ± 0.03^a^	0.45 ± 0.05^a^
Albuginea weight (g)	0.22 ± 0.13^a^	0.22 ± 0.12^a^	0.20 ± 0.08^a^	0.37 ± 0.13^a^
Parenchyma weight (g)	1.41 ± 0.19^a^	1.24 ± 0.27^a^	1.60 ± 0.19^a^	1.27 ± 0.15^a^
Epididymis (g)	0.83 ± 0.08^a^	0.86 ± 0.15^a^	0.88 ± 0.08^a^	0.77 ± 0.04^a^
Epididymis (g/100 g)	0.22 ± 0.03^a^	0.21 ± 0.03^a^	0.21 ± 0.02^a^	0.18 ± 0.02^a^
Testosterone (ng dL^−1^)	76.8 ± 2.58^a^	62.0 ± 2.73^b^	66.6 ± 4.03^b^	50.0 ± 5.70^c^

Mean ± SD

^a,b,c^Different letters on the same line indicate significant differences among the groups (p < 0.05) by Tukey’s test. (*n* = 6 animals/group)

### Micromineral analysis

**Fig. 1:**
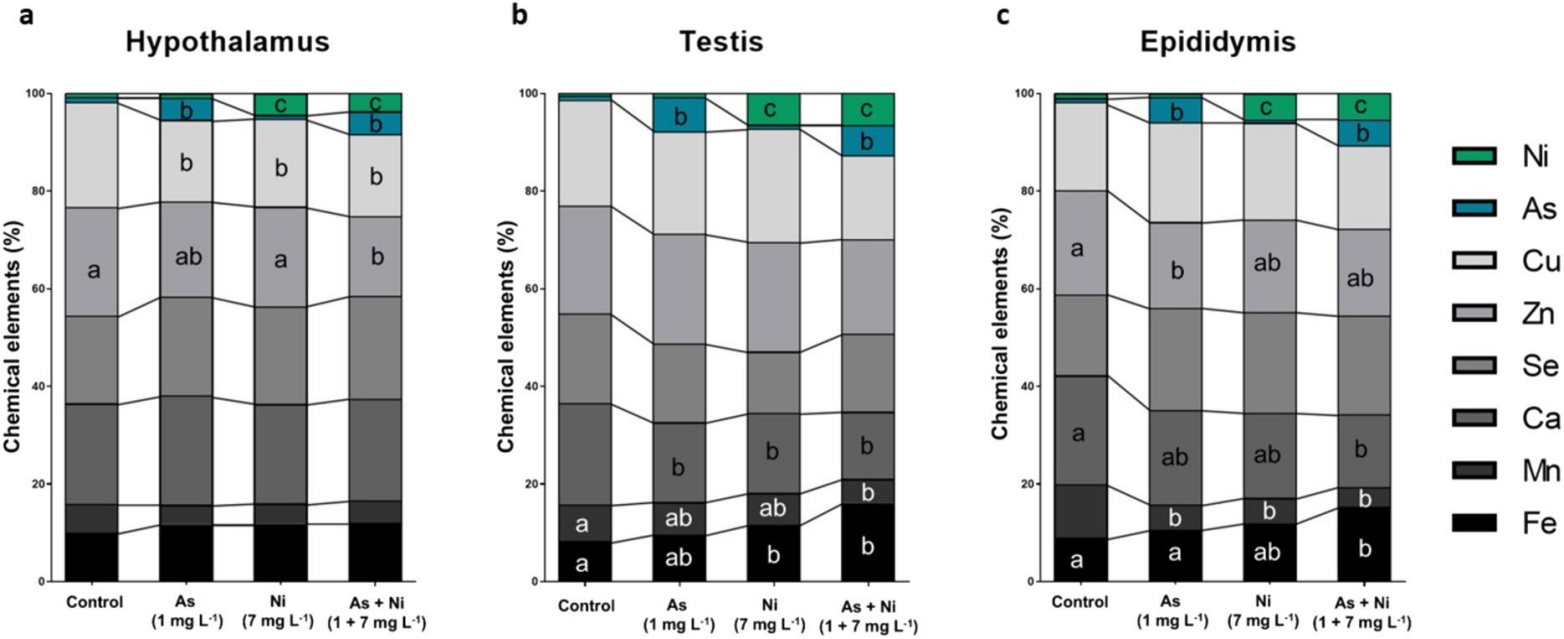
Proportion of microelements in the hypothalamus (**a**), testis (**b**) and cauda epididymis (**c**) of adult Wistar rats exposed to arsenic (As, 1 mg L^−1^) and/or nickel (Ni, 7 mg L^−1^) in drinking water for 70 days. Mean ± SD. ^a,b,c^Different letters in the same row indicate differences among the groups (p < 0.05) by Tukey’s test. (*n* = 6 animals/group)

The proportion of arsenic in the hypothalamus, testis, and epididymis was higher in rats from the As groups than in rats from the control group and Ni group (p < 0.05; Fig. 1a–c). Rats that ingested nickel solutions presented a higher proportion of this transition metal in the hypothalamus, testis, and epididymis than rats from the control and As groups (p < 0.05; Fig. 1a–c). Regarding the other microminerals, the percentage of Cu was lower in the hypothalamus of animals from the As, Ni, and As + Ni groups than in rats from the control group (p < 0.05; Fig. 1a). Moreover, the percentage of Zn was lower in animals co-exposed to arsenic and nickel than in rats from the control and Ni groups (p < 0.05; Fig. 1a). The proportion of Se, Ca, Mn, and Fe in the hypothalamus was not altered between control and exposed groups (p > 0.05; Fig. 1a). In the testis, rats from As, Ni, and As + Ni groups presented a lower percentage of Ca than control animals (p < 0.05; Fig. 1b). Rats from As + Ni group presented a lower percentage of Mn than control animals (p < 0.05; Fig. 1b). Inversely, rats from Ni and As + Ni groups presented a higher percentage of Fe than animals from the control group (p < 0.05; Fig. 1b). The proportion of Cu, Zn, and Se did not alter between control and exposed animals (p > 0.05; Fig. 1b). In the epididymis, the percentage of Zn in the animals from As group was lower than in rats from the control group (p < 0.05; Fig. 1c). The percentage of Ca in the epididymis of animals from As + Ni group was lower than in control rats (p < 0.05; Fig. 1c). Rats from As, Ni, and As + Ni groups presented a lower percentage of Mn than in rats from the control group (p < 0.05; Fig. 1c). Still, rats from As + Ni group presented a higher percentage of Fe than control animals and As-exposed rats (p < 0.05; Fig. 1c). The proportion of Cu and Se did not differ between rats from control and exposed groups (p > 0.05; Fig. 1c).

### Activity of antioxidant enzymes and markers of oxidative stress

**Fig. 2:**
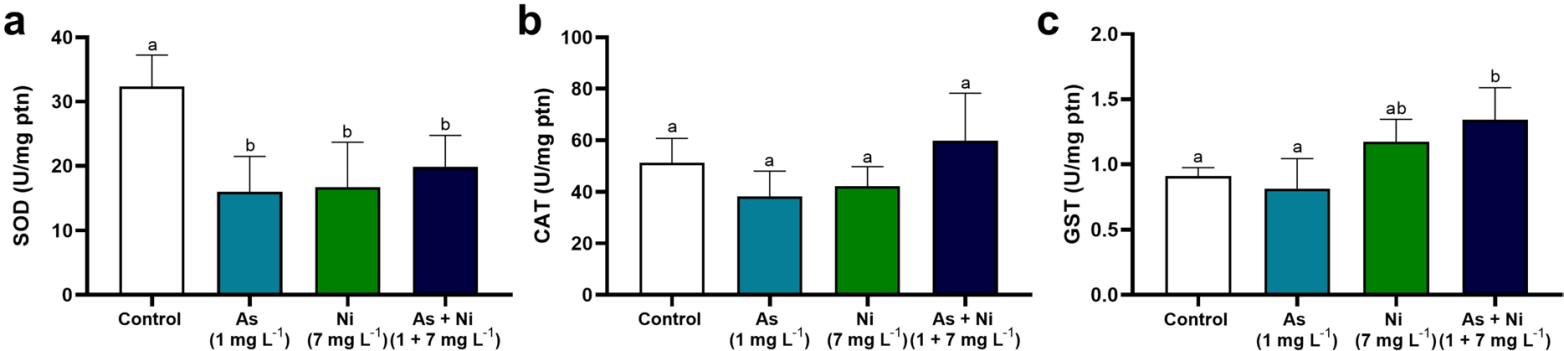
Antioxidant enzyme activity and oxidative/nitrosative markers in the hypothalamus of rats exposed to arsenic (As, 1 mg L^−1^), nickel (Ni, 7 mg L^−1^), and arsenic + nickel (As + Ni, 1 mg L^−1^ and 7 mg L^−1^, respectively) for 70 days. SOD = superoxide dismutase (**a**); CAT = catalase (**b**); GST = glutathione S-transferase (**c**). ^a,b^Different letters on the same row indicate differences among the groups (p < 0.05) by Tukey’s test. (*n* = 6 animals/group)

**Fig. 3:**
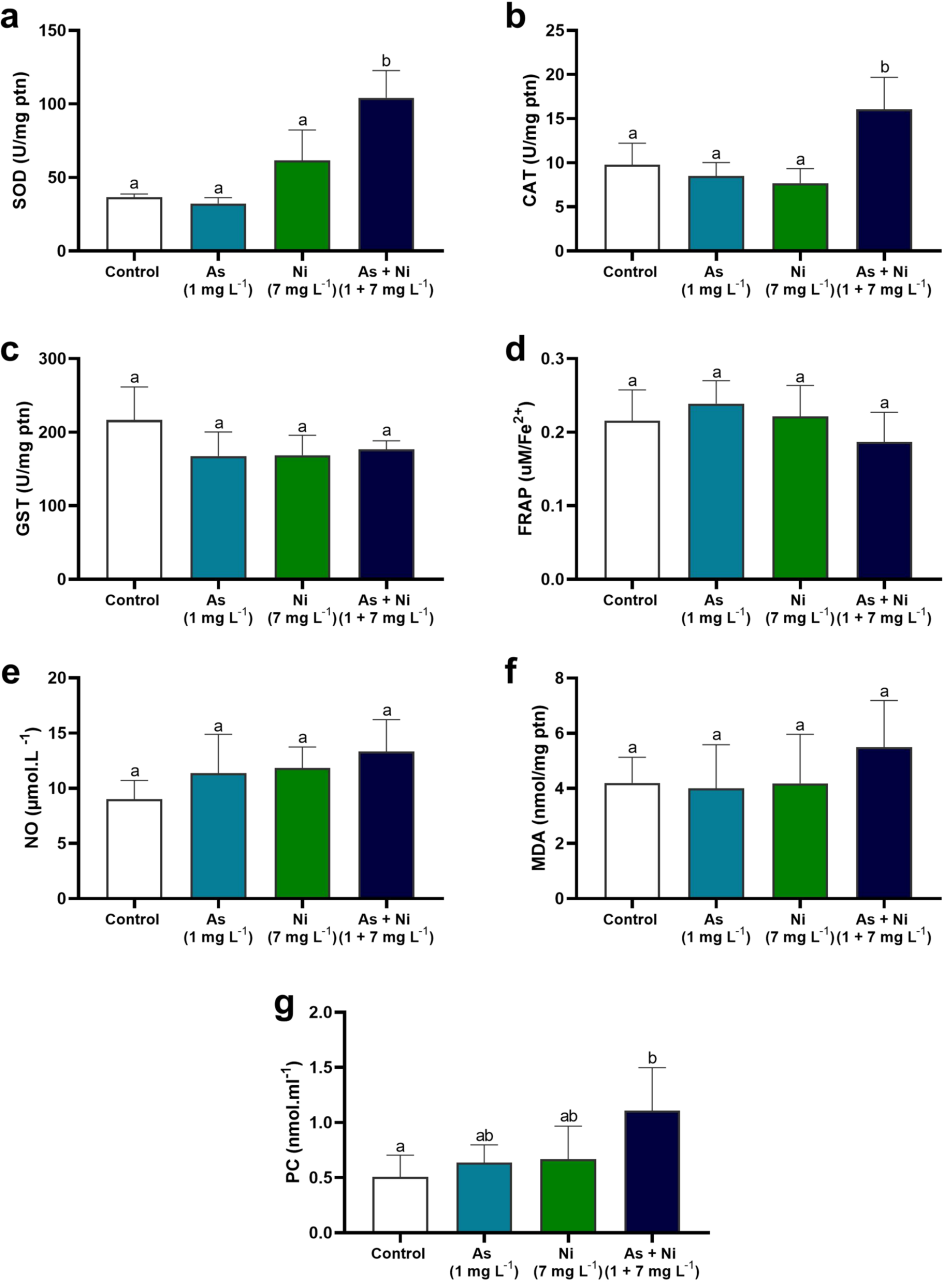
Antioxidant enzyme activity and oxidative/nitrosative markers in the testis of rats exposed to arsenic (As, 1 mg L^−1^), nickel (Ni, 7 mg L^−1^), and arsenic + nickel (As + Ni, 1 mg L^−1^ and 7 mg L^−1^, respectively) for 70 days. SOD = superoxide dismutase (**a**); CAT = catalase (**b**); GST = glutathione S-transferase (**c**); FRAP = total antioxidant capacity (**d**); NO = nitric oxide (**e**); MDA = malondialdehyde (**f**); PC = protein carbonyl (**g**). Mean ± SD. ^a,b^Different letters on the same row indicate differences among the groups (p < 0.05) by Tukey’s test. (*n* = 6 animals/group)

**Fig. 4:**
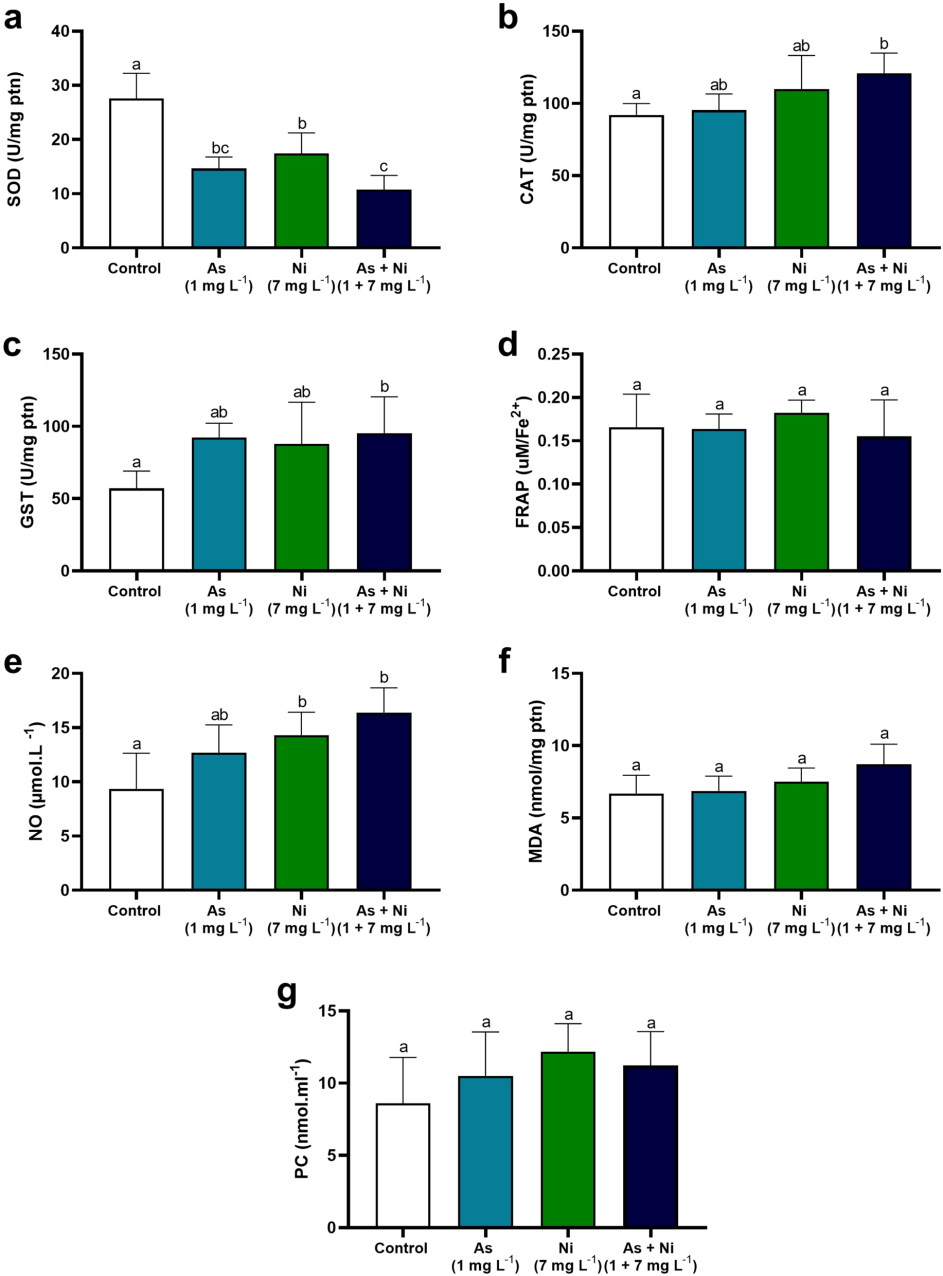
Antioxidant enzyme activity and oxidative/nitrosative markers in the cauda epididymis of rats exposed to arsenic (As, 1 mg L^−1^), nickel (Ni, 7 mg L^−1^), and arsenic + nickel (As + Ni, 1 mg L^−1^ and 7 mg L^−1^, respectively) for 70 days. SOD = superoxide dismutase (**a**); CAT = catalase (**b**); GST = glutathione S-transferase (**c**); FRAP = total antioxidant capacity (**d**); NO = nitric oxide (**e**); MDA = malondialdehyde (**f**); PC = protein carbonyl (**g**). Mean ± SD. ^a,b,c^Different letters on the same row indicate differences among the groups (p < 0.05) by Tukey’s test. (*n* = 6 animals/group)

The hypothalamus of rats from As, Ni, and As + Ni groups showed lower SOD activity than the hypothalamus of control animals (p < 0.05; Fig. 2a). Conversely, GST activity was higher in animals co-exposed to arsenic and nickel than in control rats and those exposed to arsenic only (p < 0.05; Fig. 2c). No significant change in CAT activity was observed in the hypothalamus of rats from all experimental groups (p > 0.05; Fig. 2b). In the testis, in turn, SOD and CAT activity was higher in rats from As + Ni group than in animals from the other groups (p < 0.05; Fig. 3a and b). Other oxidative parameters, including GST activity, FRAP, NO, and MDA levels, did not differ between control and metal-exposed groups (p > 0.05; Fig. 3c–f). On the other hand, PC levels were higher in the As + Ni group rats than in control rats (p < 0.05; Fig. 3g). In the epididymis, there was a decrease in SOD activity in animals in the As, Ni, and As + Ni groups compared to the control group (p < 0.05; Fig. 4a). CAT and GST activity was higher in the epididymis of As + Ni-exposed rats than in control animals (p < 0.05; Fig. 4b and c). NO levels were higher in animals from the Ni and As + Ni groups than in rats from the control group (p < 0.05; Fig. 4e). FRAP, MDA, and PC levels did not alter between rats from the experimental groups (p > 0.05; Fig. 4d, f, and g).

### Histopathological analysis

**Fig. 5:**
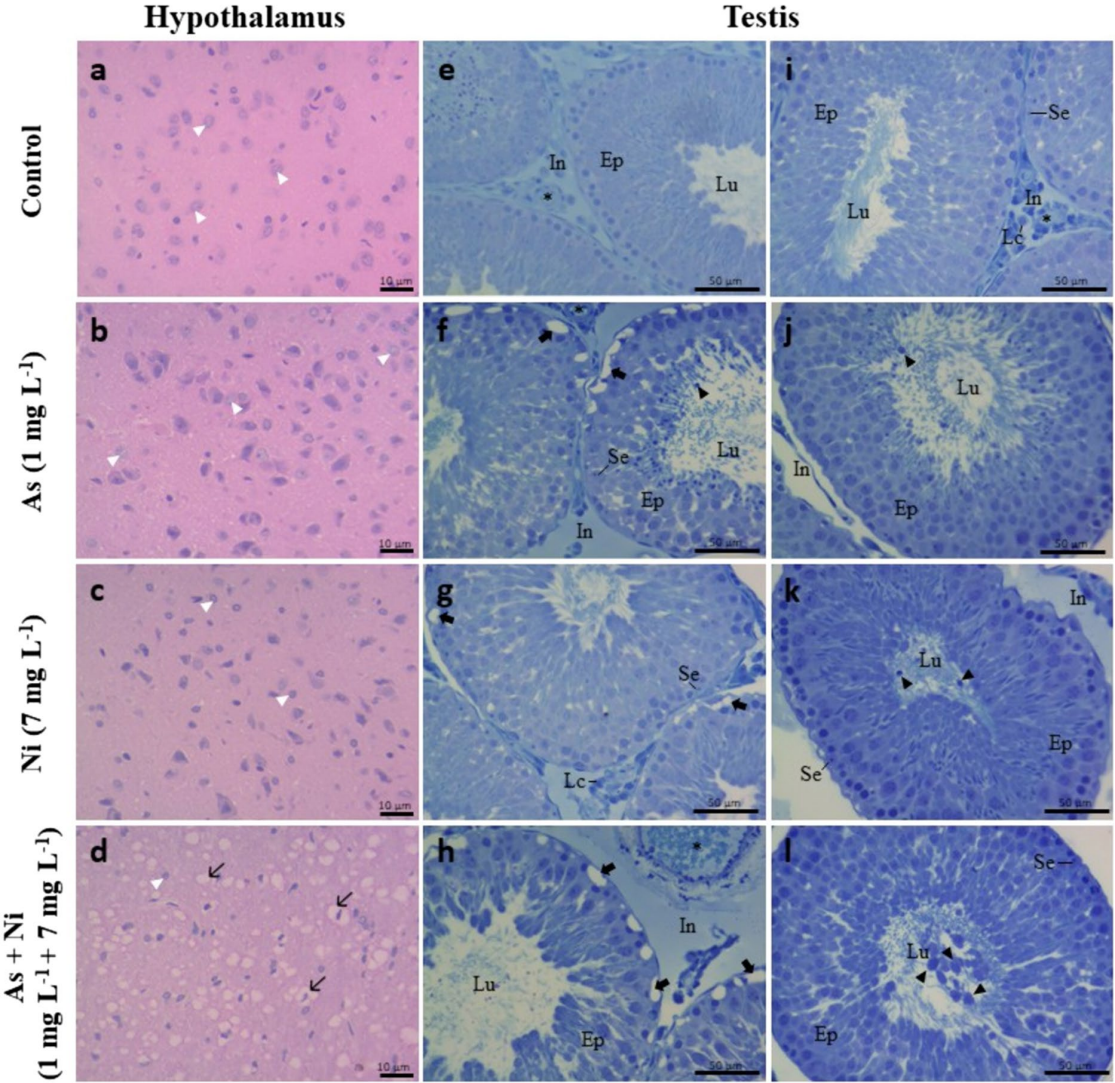
Histological sections of the hypothalamus and testis of Wistar rats exposed to arsenic (As, 1 mg L^−1^) and/or nickel (Ni, 7 mg L^−1^) in drinking water for 70 days. Figures **a**, **b**, **c**, and **d** represent the hypothalamus, showing intact neurons (white arrowhead) and degeneration (thin arrow) in panel **d**. From **e-l** are histological testis sections. *Lu* seminiferous tubule lumen, *Ep* seminiferous epithelium, *In* intertubular compartment, *Lc*, Leydig cell, * blood vessels; Se, Sertoli cell; thick arrow, vacuoles in the seminiferous epithelium; black arrowhead, germ cells in the lumen. Hematoxylin and eosin or toluidine blue staining. Scale bars = 10 μm (**a-d**) and 50 μm (**e-l**)

**Fig. 6:**
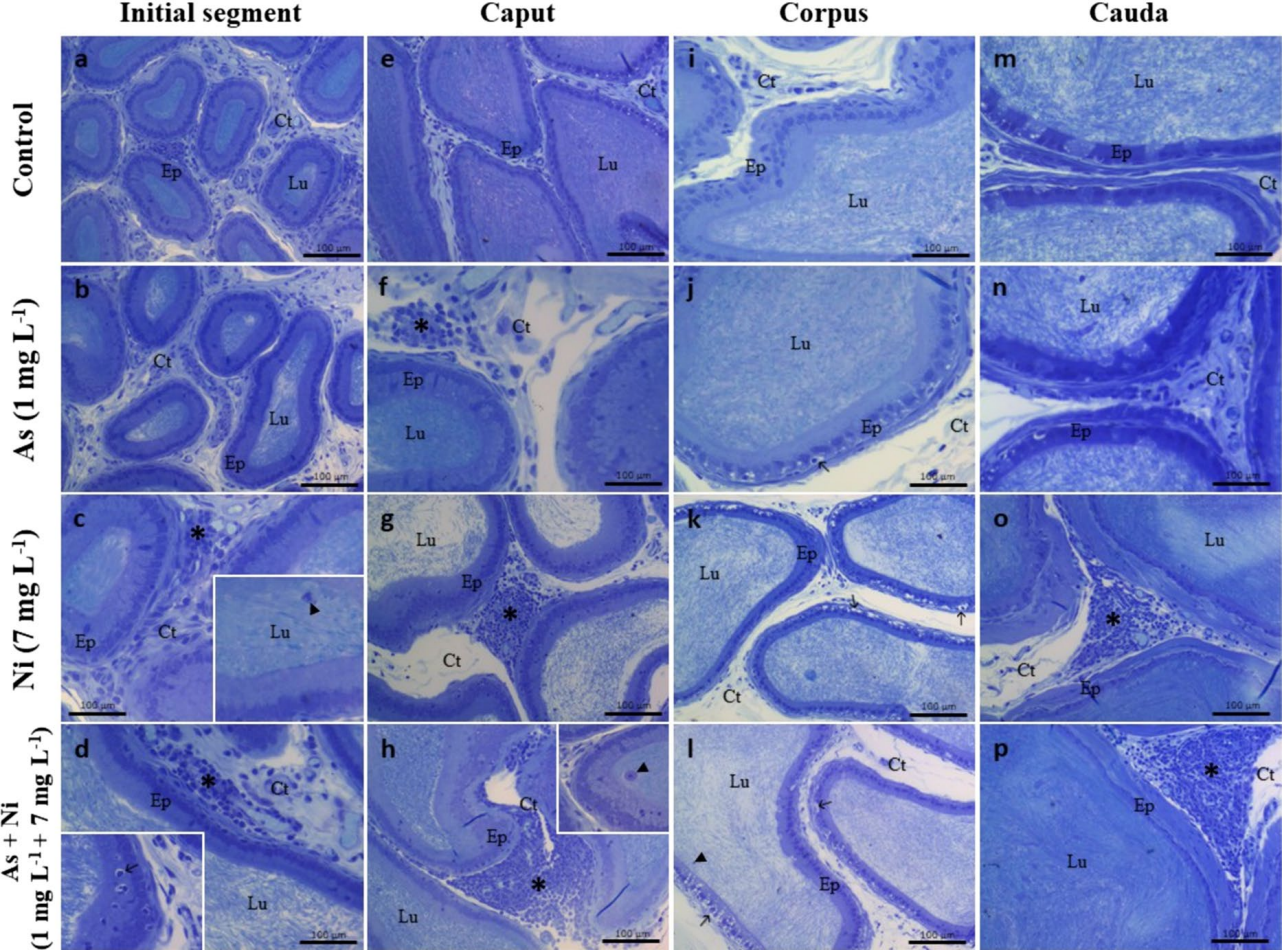
Histological sections of the epididymis of Wistar rats exposed to arsenic (As, 1 mg L^−1^) and/or nickel (Ni, 7 mg L^−1^) in drinking water for 70 days. Initial segment (**a–d**); caput (**e–h**); corpus (**i–l**); cauda (**m–p**). *Lu* seminiferous tubule lumen, *Ep* seminiferous epithelium, *Ct*, connective tissue, arrowhead, germ cells in the lumen; * inflammatory infiltrate; arrow, vacuole. Toluidine blue staining. Scale bars = 100 μm

The hypothalamus of control rats and those exposed to arsenic or nickel alone exhibited normal tissue organization with well-preserved neuronal architecture and nuclei of typical morphology. In contrast, animals co-exposed to arsenic and nickel showed focal areas of neuronal degeneration (Fig. 5), clear vacuoles in the cytoplasm, cytoplasmic retraction, and loss of typical architecture. The testes of control rats showed regular tissue architecture, comprising a tunica propria, seminiferous epithelium, and a lumen in the tubular compartment. The epithelium with Sertoli cells and layers of germ cells (Fig. 5). In addition, connective tissue, Leydig cells, lymphatic spaces, blood vessels, and macrophages were evident in the intertubular compartment (Fig. 5). There was the presence of vacuoles at the base of the epithelium in all groups, being more frequent in animals exposed to metals. In addition, germ cells were prominently present in the lumen of the seminiferous tubules of animals from As, Ni, and As + Ni groups (Fig. 5). In the epididymis, the epididymal duct of control rats presented regular tissue organization, with a single epithelial layer composed of typical cells (principal, basal, and clear cells) and a lumen filled with sperm in the regions of the initial segment, caput, corpus, and cauda epididymis (Fig. 6). In the initial segment, the presence of foci of inflammatory infiltrate and germ cells in the lumen of the duct was observed mainly in animals from Ni and As + Ni groups (Fig. 6). In the caput epididymis, the presence of inflammatory infiltrate was observed in animals from As, Ni, and As + Ni groups, in addition to the presence of germ cells in the epididymal duct of animals from As + Ni group (Fig. 6). In the corpus epididymis, vacuoles were observed at the base of the tubular epithelium in all groups, with the presence of epithelial desquamation being more frequent in rats from As + Ni group (Fig. 6). In the cauda region, there was a presence of inflammatory infiltrate in rats from As, Ni, and As + Ni groups (Fig. 6).

### Testicular morphometry and stereology

The luminal diameter of the seminiferous tubules was higher in animals from Ni and As + Ni groups than in rats from the control group (p < 0.05; Table 2). The seminiferous epithelium height was diminished in the testis of exposed rats compared to control rats (p < 0.05; Table 2). The total tubular diameter of the seminiferous tubules did not differ between rats from the experimental groups (p > 0.05; Table 2). Moreover, animals from the As + Ni group showed a lower percentage of seminiferous tubules and epithelium than animals from the other groups (p < 0.05; Table 2). Rats from the As + Ni group showed a higher luminal percentage and intertubule proportion than animals from the other groups (p < 0.05; Table 2). The percentage of tunica propria did not differ between groups (p > 0.05; Table 2).

**Table 2:** Histomorphometry and stereology of the testis from adult Wistar rats exposed to arsenic (As) and nickel (Ni) in drinking water for 70 days

Parameters	Control	1 mg L^−1^ As	7 mg L^−1^ Ni	1 mg L^−1^ As + 7 mg L^−1^ Ni
Testicular morphometry				
Tubular diameter (μm)	277.4 ± 14.49^a^	255.8 ± 10.96^a^	256.2 ± 12.56^a^	254.7 ± 17.76^a^
Luminal diameter (μm)	65.45 ± 11.59^a^	72.60 ± 8.42^ab^	85.88 ± 5.88^b^	91.15 ± 12.22^b^
Epithelium height (μm)	105.90 ± 2.48^a^	91.58 ± 6.04^b^	85.67 ± 5.30^b^	81.79 ± 12.25^b^
Volumetric proportion				
Seminiferous tubules (%)	78.61 ± 3.67^a^	75.20 ± 3.02^a^	74.41 ± 2.20^a^	64.13 ± 8.45^b^
Seminiferous epithelium (%)	69.51 ± 2.87^a^	65.62 ± 4.38^a^	64.56 ± 3.42^a^	51.72 ± 6.72^b^
Tunica propria (%)	2.53 ± 0.45^a^	2.47 ± 0.27^a^	2.46 ± 0.19^a^	2.06 ± 0.34^a^
Lumen (%)	6.56 ± 0.81^a^	7.10 ± 1.14^a^	7.38 ± 0.66^a^	10.35 ± 1.97^b^
Intertubule (%)	21.39 ± 3.69^a^	24.80 ± 3.02^a^	25.59 ± 2.20^a^	35.87 ± 8.45^b^

Mean ± SD

^a,b^Different letters on the same row indicate significant differences among the groups (*p* < 0.05) by Tukey’s test. (*n* = 6 animals/group)

Regarding the volumetric proportion of intertubular components, rats from the As + Ni group presented higher connective tissue and lymphatic space than animals from the other groups (p < 0.05; Table 3). The proportion of blood vessels was higher in animals from Ni and As + Ni groups than in control rats (p < 0.05; Table 3). The proportion of macrophages was higher in rats from the As + Ni group than in rats from the Ni group (p < 0.05; Table 3). The percentage of Leydig cells was lower in rats from Ni and As + Ni groups than in control animals (p < 0.05; Table 3). Also, rats from the As + Ni group presented a higher percentage of connective tissue and lymphatic space than animals from the other groups (p < 0.05; Table 3). The proportion of blood vessels was also higher in animals from Ni and As + Ni groups than in rats from the control group (p < 0.05; Table 3). Leydig cell volume was lower in rats from the As + Ni group than in rats from the control and As groups (p < 0.05; Table 3). Macrophage volume did not differ between groups (p > 0.05; Table 3).

**Table 3:** Volumetric proportion (%) and volume (mL) of the intertubular components in the testis from adult Wistar rats exposed to arsenic (As) and nickel (Ni) in drinking water for 70 days

Parameters	Control	1 mg L^−1^ As	7 mg L^−1^ Ni	1 mg L^−1^ As + 7 mg L^−1^ Ni
Volumetric proportion				
Connective tissue (%)*	0.80 ± 0.19^a^	1.11 ± 0.45^ab^	1.08 ± 0.16^ab^	2.29 ± 0.68^b^
Lymphatic space (%)	5.84 ± 1.19^a^	7.64 ± 1.06^a^	9.42 ± 1.33^a^	16.25 ± 4.92^b^
Blood vessel (%)	1.84 ± 0.64^a^	3.72 ± 0.83^ab^	5.26 ± 0.98^b^	7.89 ± 1.98^c^
Macrophages (%)	0.25 ± 0.17^ab^	0.20 ± 0.04^ab^	0.16 ± 0.04^a^	0.40 ± 0.08^b^
Leydig cell (%)*	10.71 ± 2.47^a^	12.11 ± 1.04^a^	9.66 ± 0.47^a^	9.03 ± 1.24^a^
Volume				
Connective tissue (mL)	0.02 ± 0.007^a^	0.03 ± 0.01^a^	0.03 ± 0.003^a^	0.06 ± 0.01^b^
Lymphatic space (mL)	0.18 ± 0.04^a^	0.25 ± 0.05^a^	0.32 ± 0.07^a^	0.47 ± 0.11^b^
Blood vessel (mL)	0.05 ± 0.02^a^	0.12 ± 0.03^ab^	0.17 ± 0.03^bc^	0.23 ± 0.04^c^
Macrophages (mL)	0.68 ± 0.31^a^	0.47 ± 0.43^a^	0.44 ± 0.16^a^	0.80 ± 0.53^a^
Leydig cell (mL)	0.40 ± 0.09^a^	0.39 ± 0.06^a^	0.33 ± 0.04^ab^	0.26 ± 0.03^b^

Mean ± SD

^a,b,c^Different letters on the same row indicate significant differences among the groups (p < 0.05) by ANOVA and Tukey’s test or *^a,b,c^ Different letters on the same row indicate significant differences among the groups (p < 0.05) by Kruskal–Wallis followed by Dunn’s test. (*n* = 6 animals/group)

Regarding the morphometry of Leydig cells, there was a decrease in the nuclear diameter and volume in animals from all exposed groups compared to the control group (p < 0.05; Table 4). Rats from the As + Ni group had a lower nuclear percentage than the nuclear percentage observed in rats from the control group (p < 0.05; Table 4). The cytoplasmic volume was lower in animals from the Ni and As + Ni groups than in their controls (p < 0.05; Table 4). The volume of Leydig cells in animals exposed to Ni and As + Ni was lower compared to the control (p < 0.05; Table 4). The cytoplasmic percentage and the number of cells/testis (× 10^6^) did not change between rats from the control and exposed groups (p > 0.05; Table 4).

**Table 4:** Leydig cells stereology of adult Wistar rats exposed to arsenic (As) and nickel (Ni) in drinking water for 70 days

Parameters of Leydig cells	Control	1 mg L^−1^ As	7 mg L^−1^ Ni	1 mg L^−1^ As + 7 mg L^−1^ Ni
Nuclear diameter (μm)	8.4 ± 0.3^a^	7.5 ± 0.5^b^	7.1 ± 0.5^b^	6.9 ± 0.6^b^
Nuclear volume (μm^3^)	314.8 ± 30.8^a^	219.3 ± 47.5^b^	190.8 ± 41.0^b^	177.7 ± 44.2^b^
Nuclear percentage (%)	2.8 ± 0.4^a^	2.18 ± 0.3^ab^	2.4 ± 0.3^ab^	1.98 ± 0.3^b^
Cytoplasmatic volume (μm^3^)	1179.0 ± 155.0^a^	1038.0 ± 394.8^ab^	592.2 ± 148.4^c^	634.5 ± 174.3^bc^
Cytoplasm percentage (%)	8.0 ± 4.7^a^	9.93 ± 1.0^a^	7.3 ± 0.3^a^	7.04 ± 1.0^a^
Cell volume (μm^3^)	1494.0 ± 176.7^a^	1257.0 ± 434.8^ab^	783.0 ± 186.8^b^	812.2 ± 216.8^b^
Number of cells/testis (× 10^6^)	282.8 ± 89.4^a^	339.7 ± 95.6^a^	444.1 ± 136.9^a^	342.3 ± 83.0^a^

Mean ± SD

^a,b,c^Different letters on the same row indicate significant differences among the groups (p < 0.05) by Tukey’s test. (*n* = 6 animals/group)

### Daily sperm production, sperm number, and sperm transit time in the epididymis

The number of spermatids per testis and daily sperm production was lower in rats from the As, Ni, and As + Ni groups than in animals from the control group (p < 0.05; Table 5). However, the number of spermatids per gram of testis did not differ between control and exposed groups (p > 0.05; Table 5). The number of sperm in the caput/corpus region was lower in rats from As, Ni, and As + Ni groups than in control animals (p < 0.05; Table 5). The transit time in the caput/corpus region was shorter in animals co-exposed to arsenic and nickel than in control rats (p < 0.05; Table 5). Similarly, the number of sperm in the cauda/organ was lower in rats from the As, Ni, and As + Ni groups compared to control animals (p < 0.05; Table 5). In the cauda epididymis, sperm transit time in animals from the As + Ni group was shorter than in control animals (p < 0.05; Table 5).

**Table 5:** Sperm count parameters in the testis and epididymis of Wistar rats exposed to arsenic (As) and nickel (Ni) in drinking water for 70 days

Parameters	Control	1 mg L^−1^ As	7 mg L^−1^ Ni	1 mg L^−1^ As + 7 mg L^−1^ Ni
Spermatid number (× 10^6^/testis)	149.9 ± 19.3^a^	113.3 ± 11.7^b^	117.1 ± 9.6^b^	103.0 ± 10.2^b^
Spermatid number (× 10^6^/g testis)	109.1 ± 30.6^a^	93.7 ± 17.7^a^	73.8 ± 11.1^a^	78.7 ± 17.7^a^
Dailly sperm production (× 10^6^/organ)	24.6 ± 2.2^a^	18.6 ± 1.9^b^	19.2 ± 1.6^b^	16.9 ± 1.7^b^
Caput/corpus epididymis sperm number (× 10^6^/ organ)	70.7 ± 3.6^a^	44.4 ± 5.7^bc^	46.3 ± 5.0^b^	30.3 ± 6.1^c^
Sperm transit time in the caput/corpus epididymis (days)	2.9 ± 0.1^a^	2.4 ± 0.5^ab^	2.4 ± 0.2^ab^	1.8 ± 1.2^b^
Cauda epididymis sperm number (× 10^6^/organ)	148.9 ± 15.4^a^	89.1 ± 6.2^b^	89.3 ± 4.7^b^	69.6 ± 3.4^b^
Sperm transit time in the cauda epididymis (days)	6.2 ± 1.7^a^	4.8 ± 0.8^ab^	4.7 ± 0.79^ab^	4.15 ± 0.5^b^
Sperm motility (%)	86.0 ± 1.2^a^	70.8 ± 0.8^b^	66.2 ± 8.4^b^	59.2 ± 5.6^b^

Mean ± SD

^a,b,c^Different letters on the same row indicate significant differences among the groups (*p* < 0.05) by Tukey’s test. (*n* = 6 animals/group)

### Sperm motility and morphology

**Fig. 7:**
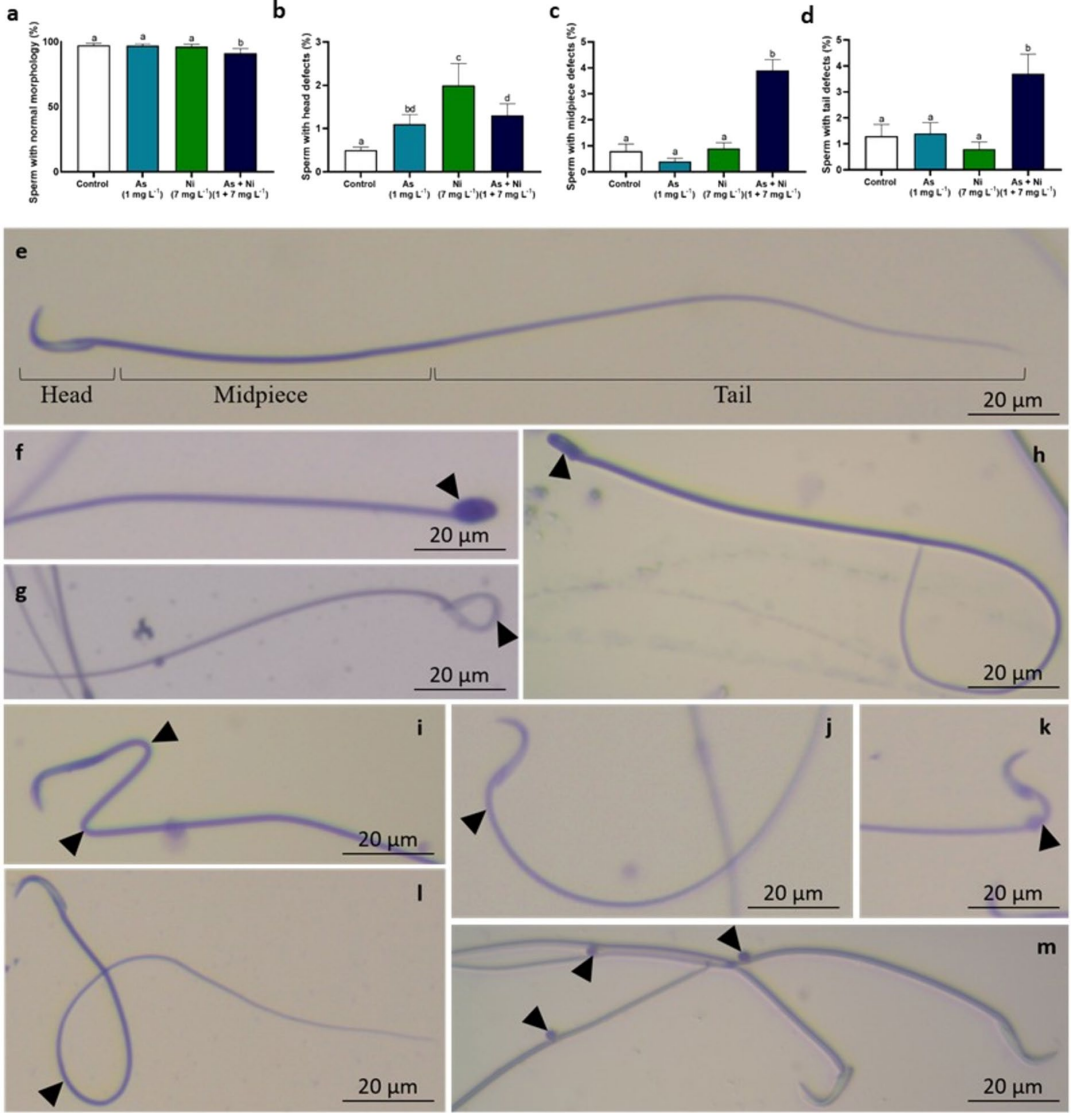
Sperm morphology of adult Wistar rats exposed to arsenic (As, 1 mg L^−1^) and/or nickel (Ni, 7 mg L^−1^) in drinking water for 70 days. Sperm with normal morphology (%) (**a**); Sperm with head defects (%) (**b**); Sperm with midpiece defects (%) (**c**); Sperm with tail defects (%) (**d**). Sperm with normal morphology (**e**); Sperm with defective morphology (arrowhead) (**f-m**). Mean ± SD. ^a,b,c,d^Different letters in the same row indicate differences among the groups (p < 0.05) by Tukey’s test. (*n* = 6 animals/group)

Moreover, sperm motility was lower in rats from the As, Ni, and As + Ni groups than in control animals (p < 0.05; Table 5). Rats co-exposed to arsenic and nickel exhibited a lower percentage of sperm with normal morphology than rats from the other groups (p < 0.05; Fig. 7). The percentage of sperm with head defects was higher in animals from the As, Ni, and As + Ni groups than in rats from the control group (p < 0.05; Fig. 7). The percentage of sperm with defects in the midpiece and tail was higher in animals co-exposed to arsenic and nickel than rats from the other groups (p < 0.05; Fig. 7).

## Discussion

Male reproductive damage caused by environmental pollutants, such as arsenic and nickel, is well-documented. However, the interaction of these elements is unclear, especially regarding their effects on male reproductive morphophysiology, as they can play synergistic, additive, or antagonistic roles (Groten et al. 2001; Adedara et al. 2017). The current study provides pioneering data on the combined exposure to environmentally relevant doses of arsenic and nickel in water and its consequences on the hypothalamus, testis, epididymis, and sperm parameters in adult Wistar rats. Such experimental design becomes relevant and of ecotoxicological interest since humans and animals are usually exposed to mixtures of chemical elements rather than isolated ones (Sani et al. 2023). Herein, exposure to arsenic and nickel affected the hypothalamus, testis, and epididymis, compromising the organs’ redox state. Moreover, our results showed high metal retention in these organs, disturbing the homeostasis of trace elements and triggering initial damage due to hormonal changes, histological alterations, and nitrosative and oxidative events in the testis and epididymis, with no alteration in biometric parameters. Altogether, these changes may compromise sperm production, number, morphology, motility, and transit time in the epididymis.

Co-exposure to arsenic and nickel caused neuronal degeneration in the tissular architecture of the hypothalamus. This alteration may have occurred due to the combination of metal retention, breakdown of trace element homeostasis, and dysregulation of antioxidant enzymes. Overall, the co-exposure of arsenic and nickel, isolated or combined, may create an oxidative environment due to a dysregulation of SOD and GST activity. Typically, these enzymes scavenge or suppress the formation of reactive oxygen and nitrogen species (Ganger et al. 2016). While SOD is the enzyme that converts superoxide anions into less harmful substances, such as hydrogen peroxide, GST acts in cellular detoxification by neutralizing hydrogen peroxide and catalyzing the conjugation of glutathione to electrophilic compounds, rendering them less toxic and more easily excreted (Dasari et al. 2018). Herein, the low SOD activity observed in rats from all exposed groups may reflect the high presence of superoxide anions within the hypothalamic tissue due to the exhaustion of this enzyme (Chidambaram et al. 2024) and low hydrogen peroxide generation, which maintained CAT activity. In co-exposed rats, the overload of superoxide anions may be involved in the focal areas of degeneration observed in their hypothalamus. The resulting redox imbalance promotes lipid peroxidation, protein modification, and neuronal DNA damage, processes that can trigger the activation of pro-apoptotic pathways, such as the caspase-3 pathway, which has already been described in models exposed to arsenic (Adedara et al. 2019b; Vázquez Cervantes et al. 2023) and nickel (Yang et al. 2021). Moreover, the high activity of GST observed in co-exposed rats evidenced their synergic effect, intensifying the detoxification process of the hypothalamus through the glutathione pathway.

Another biochemical alteration that may contribute to the tissue damage in the hypothalamus was the low proportion of Cu observed in rats exposed to arsenic and nickel, isolated and combined, besides the low concentration of Zn in co-exposed rats. Copper and Zn are crucial cofactors of antioxidant enzymes, including SOD (Oteiza et al. 1999; Souza et al. 2019; Chen et al. 2023), and are relevant to the hypothalamus’ functioning. For instance, Cu and Zn maintain the stability of hypothalamic granules containing luteinizing hormone-releasing hormone (GnRH) and stimulate its release (Martin and White 1992; Herman et al. 2012). Once GnRH regulates metabolic and neuroendocrine mechanisms essential for the hypothalamic-pituitary-gonadal axis due to its role in the pituitary gland functioning (Maggi et al. 2016; Dhole and Kumar 2017), a reduction in its release may negatively affect the secretion of FSH and LH (Kong et al. 2014; Bashandy et al. 2016; Adedara et al. 2019a; Wu et al. 2020).

Furthermore, the proportion of arsenic and nickel, obtained after an isolated or simultaneous exposure, was remarkable in the rat testis. The presence of both toxic metals within the testis elevated Fe and diminished Mn, whereas Ca was lower in rats from all exposed groups. Arsenic and nickel exhibit ionic mimicry with trace elements, including Ca and Fe, competing for binding sites on membrane transport proteins, such as divalent metal transporters and Ca channels (Valko et al. 2005; Yan et al. 2023). In the testis, arsenic- and nickel-induced dysfunction in Ca homeostasis may impair intracellular signaling vital for spermatogenesis and the function of Sertoli and Leydig cells. At the same time, Fe may be involved in pathways that amplify oxidative stress. These interaction and competition mechanisms may result from specific interactions between these chemical elements. For example, arsenic can interfere with Ca homeostasis by replacing phosphates in enzymatic reactions and impairing intracellular Ca^2+^ storage, altering cellular signaling and mitochondrial function (Guidarelli et al. 2019), while nickel can compete with Ca for cellular channels and transporters, in addition to replacing Ca^2+^ in binding proteins, interfering with intracellular signaling and epithelial stability (Forgacs et al. 2012). In the case of Fe, nickel has a high affinity for divalent metal transporters, such as divalent metal transporter 1 (DMT1), competing directly with Fe and altering its absorption and distribution (Davidson et al. 2005). Arsenic, in turn, alters Fe metabolism, negatively modulating the expression of proteins such as ferroportin, and favoring tissue accumulation (Li et al. 2025). Excessive Fe content can amplify local oxidative stress through the Fenton reaction, generating highly toxic reactive species (Valko et al. 2005; Jomova et al. 2011). Finally, low testicular Mn may compromise mitochondrial activities, including the activity of mitochondrial (Fe/Mn) SOD (Aitken and Roman 2008) and steroidogenic acute regulatory (StAR) protein (Cheng et al. 2003), and impair sperm chromatin integrity (Marzec-Wróbleska et al. 2012).

In light of the foregoing, the disruption of trace elements imbalance was one of the potential mechanisms of arsenic and nickel here, mainly after a simultaneous contamination. Trace element homeostasis is regulated by a complex system of absorption, transport, storage, and excretion, which can be affected by exposure to toxic metals. Hence, metal exposure can compromise the bioavailability of these elements by competing for cellular transporters, interfering with intestinal absorption, or promoting their excessive excretion (Ibrahim et al. 2020; Zhu et al. 2024). Arsenic and nickel compete with essential minerals, such as Fe, Ca, Zn, Cu, and Mn, for shared cellular transporters and specific ion channels (Davidson et al. 2005; Valko et al. 2005; Yan et al. 2023). This competition can lead to reduced bioavailability and intestinal absorption of these elements. Moreover, these metals can interfere with the gene expression of transport proteins, altering their tissue distribution (Li et al. 2025). Arsenic and nickel accumulation can also negatively impact intracellular storage proteins such as ferritin and metallothionein, affecting the cellular capacity to buffer free ions and increasing susceptibility to oxidative stress (Wu et al. 2006; Flora 2011). Together, these mechanisms help explain the dysregulation of trace elements observed, as well as their association with antioxidant dysfunction and reproductive impairment.

The breakdown of trace element homeostasis in the testis, along with the retention of arsenic and nickel, triggered oxidative damage by an increment in SOD and CAT activity in co-exposed animals. Essential elements, including Mn, Zn, and Cu, are fundamental cofactors for SOD activity, whereas Fe is associated with CAT function (Blokhina et al. 2003; Soetan et al. 2010). These enzymes probably increased their activity in an attempt to neutralize excess free radicals. However, when the production of reactive species exceeds cellular antioxidant capacity, it culminates in oxidative stress (Lugrin et al. 2014). Arsenic and nickel are known to cause this imbalance through distinct mechanisms (Su et al. 2011; Guvvala et al. 2016; Lima et al. 2018; Parveen et al. 2023). Arsenic, when interacting with intracellular thiols, generates reactive oxygen species (ROS) through mitochondrial dysfunction, activating the NADPH oxidase (NOX) pathway and increasing lipid peroxidation (Barbosa et al. 2010; Zhang et al. 2023). The metalloid can activate the mitogen-activated protein kinase (MAPK) pathway, promoting the transcription of pro-inflammatory and pro-apoptotic genes (Medda et al. 2021). In addition to stimulating the production of free radicals by interacting with sulfhydryl groups, nickel interferes with iron homeostasis and intensifies the formation of hydroxyl radicals via the Fenton reaction (Das et al. 2008; Sharma et al. 2023). Moreover, nickel also activates the nuclear transcription factor kappa B (NF-κB), leading to the expression of inflammatory cytokines and promoting a cellular environment more prone to oxidative damage (Guo et al. 2019).

Arsenic and nickel accumulation creating oxidative environment elicited damage to testis tissue of exposed rats from all experimental groups, with the presence of vacuoles in the seminiferous epithelium and germ cells detached in the lumen, suggesting a Sertoli cell reaction against metal and free radicals’ overload (Sun et al. 2011; Souza et al. 2016; Sen et al. 2019). Vacuole formation may indicate a cellular impairment due to oxidative damage, while germ cell detachment may be associated with structural vulnerability and inadequate androgen-mediated modulation of cell junctions (Zhang et al. 2005; Souza et al. 2019). The consequence was observed in histomorphometric and stereological parameters obtained from the tubular compartment, such as low epithelium height along with high luminal diameter with no change in tubular diameter, and volumetric proportion of tubular and intertubular components in the testis of co-exposed rats. Collectively, these findings indicate an early stage of arsenic and nickel toxicity. Indeed, morphometric analysis often identifies microscopic differences not observed by qualitative analysis under light microscopy (Machado-Neves 2022). Alterations in the morphometry of the seminiferous epithelium are related to the reduction of spermatid number and daily sperm production in exposed rats, which may be considered initial, as the spermatid number per gram of testis did not alter. As sperm precursor cells, spermatids are crucial for the continuity of spermiogenesis and spermiation, and their reduction directly compromises the reproductive potential of animals (Tesarik et al. 1998; Sanabria et al. 2015). Studies indicate that arsenic can disrupt the differentiation of round spermatids into elongated ones, aggravating the deleterious effects on gametogenesis (Han et al. 2020). Meanwhile, nickel impairs spermatogenesis by inducing testicular lesions, decreasing the number of spermatogenic cells, and affecting sperm concentration (Guo et al. 2023).

The low proportion of tubular components was compensated by the expansion of interbular components, with a high percentage of collagenous fibers, blood vessels, and lymphatic spaces in co-exposed rats. For instance, the increase in blood vessels and lymphatic space may be related to both a compensatory mechanism for the elimination of toxic substances (da Silva et al. 2021). Interestingly, the proportion of macrophages did not differ between the experimental groups. Testicular macrophages can promote extracellular matrix deposition and testicular fibrosis through the activation of profibrotic pathways, such as transforming growth factor-β1 (TGF-β1) and platelet-derived growth factor (PDGF), favoring the increase in connective tissue (Wynn and Barron 2010). These immune cells can also interfere with testosterone synthesis by releasing pro-inflammatory cytokines and ROS, inhibiting Leydig cell function (Hales 2002; Chi et al. 2024). In the present study, stereological analysis evidenced low nuclear diameter and volume in Leydig cells in all exposed rats, cytoplasmic volume and cell volume in nickel- and arsenic-nickel-exposed animals, and nuclear percentage in rats exposed simultaneously to these metals. These results indicate a decline in Leydig cells’ functionality and a maintenance of their testicular population after arsenic and nickel exposure. Studies have documented that arsenic and nickel reduce the activity of steroidogenic enzymes, such as 3ß-hydroxysteroid dehydrogenase (3ß-HSD) and 17ß-hydroxysteroid dehydrogenase (17ß-HSD; Sarkar et al. 2003; Jana et al. 2006; Yang et al. 2021; Gan et al. 2019), reducing the hormone synthesis (Payne and Hales 2004). These toxic metals can also promote Leydig cell atrophy, resulting in decreased serum testosterone levels (Jargar et al. 2012; Han et al. 2018; Noshy et al. 2022). Low testosterone levels may compromise the adhesion of round spermatids to Sertoli cells, leading to premature detachment into the lumen of the seminiferous tubules and impairing spermiogenesis (Li et al. 2024). The androgen receptor signaling pathway can also be modulated by exposure to heavy metals, affecting the function of Sertoli and Leydig cells and compromising testosterone production. This effect is mediated by direct interactions of the metals with the receptors and/or with the enzyme system involved in the synthesis of sex hormones (Reddy et al. 2011; Kong et al. 2014; Ajibade et al. 2020; Khezri Motlagh et al. 2022). Herein, we observed a reduction in serum testosterone with discrete tubular lesions in seminiferous tubules. This fact may suggest that the initial disturbance of androgen synthesis caused by arsenic and nickel exposure affected serum testosterone levels but did not impair the androgen environment within testicular parenchyma. Our hypothesis may be confirmed by the maintenance of testis weight and epididymis weight, which are androgen-dependent organs. Testosterone concentration in the testis is 100 times higher than in the serum due to the activity of androgen-binding proteins produced by Sertoli cells to warrant androgen levels to support spermatogenesis (Agarwal et al. 2003; Hess and França 2008; Machado-Neves 2022).

In the current study, the epididymis of exposed rats exhibited arsenic and nickel accumulation, disturbances in trace elements balance, mainly after co-exposure to these two metals, and antioxidant enzyme dysregulation, culminating in nitrosative damage. Altogether, these findings caused an initial alteration in epididymis histology, with the presence of inflammatory infiltrates in the interductal compartment, discrete epithelial vacuolization and desquamation, besides germ cells in the luminal duct, a consequence of their detachment in the testis, especially in animals co-exposed to arsenic and nickel. Similar to testis, epididymal SOD activity was lower in all exposed rats. Moreover, an increase in CAT and GST activity was observed in the epididymis of co-exposed animals. The imbalance between antioxidant capacity and free radical production is reflected in high NO levels in the cauda epididymis. Excess NO can react with O₂⁻ to form peroxynitrite (ONOO^−^), a highly reactive species that causes nitrosative damage, leading to the modification of proteins, lipids, and DNA (Wang et al. 2021). This damage can compromise cellular functionality, exerting nitrosative modifications in epididymal proteins (O’Flaherty 2019), potentially negatively influencing sperm motility, as observed here. ONOO^−^ can also activate the nuclear factor erythroid 2-related factor 2 (Nrf2) signaling pathway, promoting the expression of antioxidant genes. However, this adaptive response may be insufficient in chronic exposure to heavy metals (Jomova et al. 2025). This toxic environment may have influenced the low percentage of normal sperm morphology and sperm midpiece and tail defects after co-exposure to arsenic and nickel. While the midpiece has mitochondria producing energy for motility, the tail contains structures essential for sperm propulsion (Piomboni et al. 2012). Finally, we observed a reduction in sperm transit time in the caput/corpus and cauda epididymis in co-exposure rats. Sperm transit time is crucial for gamete maturation, as sperm acquire motility and fertile capacity during their passage through the epididymal duct and are exposed to the luminal microenvironment (Fernandez et al. 2008; Breton et al. 2019). High smooth muscle cell contraction in the epididymal duct promotes accelerated sperm movement in the epididymal duct (Kempinas and Klinefelter 2015). When the sperm transit time is reduced, it reduces the sperm exposure to the luminal environment and compromises their maturation, resulting in sperm structural and functional alterations (Fernandez et al. 2008; Barrachina et al. 2022).

## Conclusion

Our results revealed a synergistic interaction between arsenic and nickel, enhancing their toxicity in the hypothalamus and male reproductive organs of Wistar rats. Overall, subchronic co-exposure to both metals occurring through the oral route disrupted mineral homeostasis and redox balance and triggered a subclinical toxicity in male reproductive organs. The combined exposure to arsenic and nickel reduced serum testosterone levels, elicited oxidative stress in testis and epididymis tissues, and caused initial histological damage in the hypothalamus, testis, and epididymis compared to the effects of arsenic or nickel exposure. Negative consequences to the male gametes, such as low daily sperm production, motility, and normal morphology, reinforced the negative effect of arsenic and nickel co-exposure on male fertility. The observed bioaccumulation and associated dysregulation of trace elements essential for antioxidant defense and spermatogenesis further illustrate the multifactorial nature of their combined toxicity. These findings provide experimental evidence for understanding the complex biological effects of mixed metal exposure on male reproductive health.

## Data Availability

No datasets were generated or analysed during the current study.
